# Elimination of the flavodiiron electron sink facilitates long-term H_2_ photoproduction in green algae

**DOI:** 10.1186/s13068-019-1618-1

**Published:** 2019-12-05

**Authors:** Martina Jokel, Valéria Nagy, Szilvia Z. Tóth, Sergey Kosourov, Yagut Allahverdiyeva

**Affiliations:** 1 Molecular Plant Biology, Department of Biochemistry, University of Turku, 20014 Turku, Finland; 2 Institute of Plant Biology, Biological Research Centre, Szeged, Temesvári krt. 62, Szeged, 6726 Hungary

**Keywords:** Biohydrogen, Calvin–Benson–Bassham cycle, Flavodiiron proteins, Photosynthesis

## Abstract

**Background:**

The development of renewable and sustainable biofuels to cover the future energy demand is one of the most challenging issues of our time. Biohydrogen, produced by photosynthetic microorganisms, has the potential to become a green biofuel and energy carrier for the future sustainable world, since it provides energy without CO_2_ emission. The recent development of two alternative protocols to induce hydrogen photoproduction in green algae enables the function of the O_2_-sensitive [FeFe]-hydrogenases, located at the acceptor side of photosystem I, to produce H_2_ for several days. These protocols prevent carbon fixation and redirect electrons toward H_2_ production. In the present work, we employed these protocols to a knockout *Chlamydomonas reinhardtii* mutant lacking flavodiiron proteins (FDPs), thus removing another possible electron competitor with H_2_ production.

**Results:**

The deletion of the FDP electron sink resulted in the enhancement of H_2_ photoproduction relative to wild-type *C. reinhardtii*. Additionally, the lack of FDPs leads to a more effective obstruction of carbon fixation even under elongated light pulses.

**Conclusions:**

We demonstrated that the rather simple adjustment of cultivation conditions together with genetic manipulation of alternative electron pathways of photosynthesis results in efficient re-routing of electrons toward H_2_ photoproduction. Furthermore, the introduction of a short recovery phase by regular switching from H_2_ photoproduction to biomass accumulation phase allows to maintain cell fitness and use photosynthetic cells as long-term H_2_-producing biocatalysts.

## Background

The combustion of molecular hydrogen (H_2_) generates water as the only product; therefore, it is considered the most appealing carbon-free fuel and energy carrier. Currently, H_2_ is produced from hydrogen-rich molecules such as hydrocarbons (e.g., by steam reforming) and H_2_O (by electrolysis). The exploitation of biologically produced H_2_ is an attractive and promising approach [1–3]. Several microalgae, including *Chlamydomonas reinhardtii*, are able to use solar energy and photoproduce H_2_ via hydrogenase enzymes, which are functionally coupled with photosynthetic light reactions. The theoretical light energy to H_2_ energy conversion efficiency (LHCE) is about 10–13% in green algae [4, 5]. However, the experimental LHCE in suspension cultures is usually below 0.5% [6], and under specific conditions, LHCE as high as 1.7–4% can be reached [7–9].

*C. reinhardtii* is one of the most studied microalga species in regard to H_2_ metabolism [10, 11]. It possesses two genes encoding [FeFe]-hydrogenases: HYDA1 is the major isoform, whereas HYDA2 exhibits only 25% H_2_ production activity of HYDA1 [12]. There are three different pathways directing electrons toward the [FeFe]-hydrogenases via ferredoxin, of which two are connected with the photosynthetic electron transport (PET) chain and thus are light-dependent. The direct pathway involves photosystem (PS) II-dependent water photolysis [13]. The indirect pathway bypasses PSII, feeding [FeFe]-hydrogenases with electrons originating from starch, protein or lipid breakdown and arriving at the PQ pool via NADPH-dehydrogenase (NDA2) [14, 15]. The third pathway, named as fermentative pathway, functions under dark anaerobic conditions and involves electron transfer via a pyruvate–ferredoxin-oxidoreductase from pyruvate to the [FeFe]-hydrogenase [16, 17]. The direct PSII-dependent pathway is the preferred one regarding H_2_ photoproduction, since it occurs at the highest rate (up to 300 µmol mg Chl^−1^ h^−1^, see [18] for review). However, H_2_ photoproduction via the PSII-dependent pathway lasts only for a few minutes due to the inhibition of the hydrogenases by photosynthetically produced O_2_ [8, 19]. Besides being an inhibitor of hydrogenases, O_2_ also acts as a substrate for several enzymatic processes, e.g., for flavodiiron proteins (FDPs) [20–23].

*C. reinhardtii* possesses two FDPs (FLVA and FLVB) that likely function as heterodimers and have been demonstrated to catalyze O_2_ photoreduction during the photosynthetic induction after dark–light [24, 25] or low-to-high light transitions [26]. These proteins act as strong electron sinks downstream of PSI and are the major player enabling cell growth under fluctuating light intensities [26]. It is important to note that in a long-term FDP-mediated O_2_ photoreduction does not compete with CO_2_ fixation [24, 27]. The electron donor of FDPs in *C. reinhardtii* is still under question. To the best of our knowledge all the in vitro assays performed with recombinant *Synechocystis* FDPs show some affinity to NADPH and/or NADH [20, 28]. However, these studies did not test reduced ferredoxin or ferredoxin-NADP^+^ reductase (FNR) as potential FDP substrates. Importantly, an interaction of *C. reinhardtii* FLVB with ferredoxin 1 (FDX1) [29] and *Synechocystis* Flv1 and Flv3 with ferredoxins [30, 31] was reported and hypothesized [32] previously. Therefore, further studies are necessary in order to determine the exact electron donor to FDPs in *C. reinhardtii*. Since FDP-mediated O_2_ photoreduction and [FeFe]-hydrogenase-dependent H_2_ photoproduction take place at the same spot of the PET chain, downstream of PSI, competition for photosynthetic electrons between these two enzymes is likely to occur. However, the O_2_ dependency of FDPs and the extreme sensitivity of the [FeFe]-hydrogenases to O_2_ render this competition questionable. In this light, FDPs were also proposed to contribute to the maintenance of microoxic conditions inside the chloroplast, necessary for the [FeFe]-hydrogenase to function [33].

Most of the strategies employed in the past to obtain sustainable H_2_ photoproduction in *C. reinhardtii* followed a common concept. The photosynthetic activity and thus O_2_ evolution have to be reduced, and respiratory processes have to be increased in the cell to establish the anoxic or microoxic conditions to induce H_2_ production. The most widely accepted method to induce H_2_ production is a two-stage sulfur (S)-deprivation protocol enabling temporary separation of H_2_ photoproduction and photosynthetic O_2_ production in *C. reinhardtii* [34]. S-deprivation induces the ascorbate-driven inactivation of the O_2_-evolving complex [35], degradation of the PSII reaction centers, thus reducing photosynthetic activity, accumulation of starch and the establishment of anoxia that subsequently enables H_2_ photoproduction [34, 36].

Recently, novel methods were developed, which do not impose severe stress to the cells and their LHCEs are relatively high [3]. The so-called pulse illumination protocol sustains efficient H_2_ photoproduction in *C. reinhardtii* through a train of strong and short white light pulses superimposed over darkness or low-light background under nutrient replete conditions [8]. The protocol (i) prevents activation of the Calvin–Benson–Bassham (CBB)-cycle, (ii) hinders the accumulation of O_2_ and (iii) directs the photosynthetic electron transport to the hydrogenases. Other efficient H_2_ production protocols prevent activation of the CBB-cycle via substrate limitation (by omitting CO_2_ and acetate) under continuous illumination [9] and/or involve application of O_2_ scavengers [9, 37].

In this work, the novel pulse illumination protocol has revealed significantly higher H_2_ photoproduction yields in the FDP knockout mutant as compared to the parental strain. In this context, we investigated the role of FDPs during the anaerobic induction of photosynthesis and its possible impact on H_2_ photoproduction. By simultaneously monitoring H_2_ and O_2_ in both algal strains, we proved that FDPs and the [FeFe]-hydrogenase function simultaneously and directly compete for photosynthetic electrons. Furthermore, we demonstrated that the extension of light pulses from 1 to 6 s in the pulse sequence with the regular 9-s dark phase induces CO_2_ fixation via the CBB-cycle and that FDPs are critical for the fast induction of CO_2_ fixation.

## Results

### The *flv* knockout mutant displays increased photoautotrophic H_2_ production under pulse illumination

**Fig. 1:**
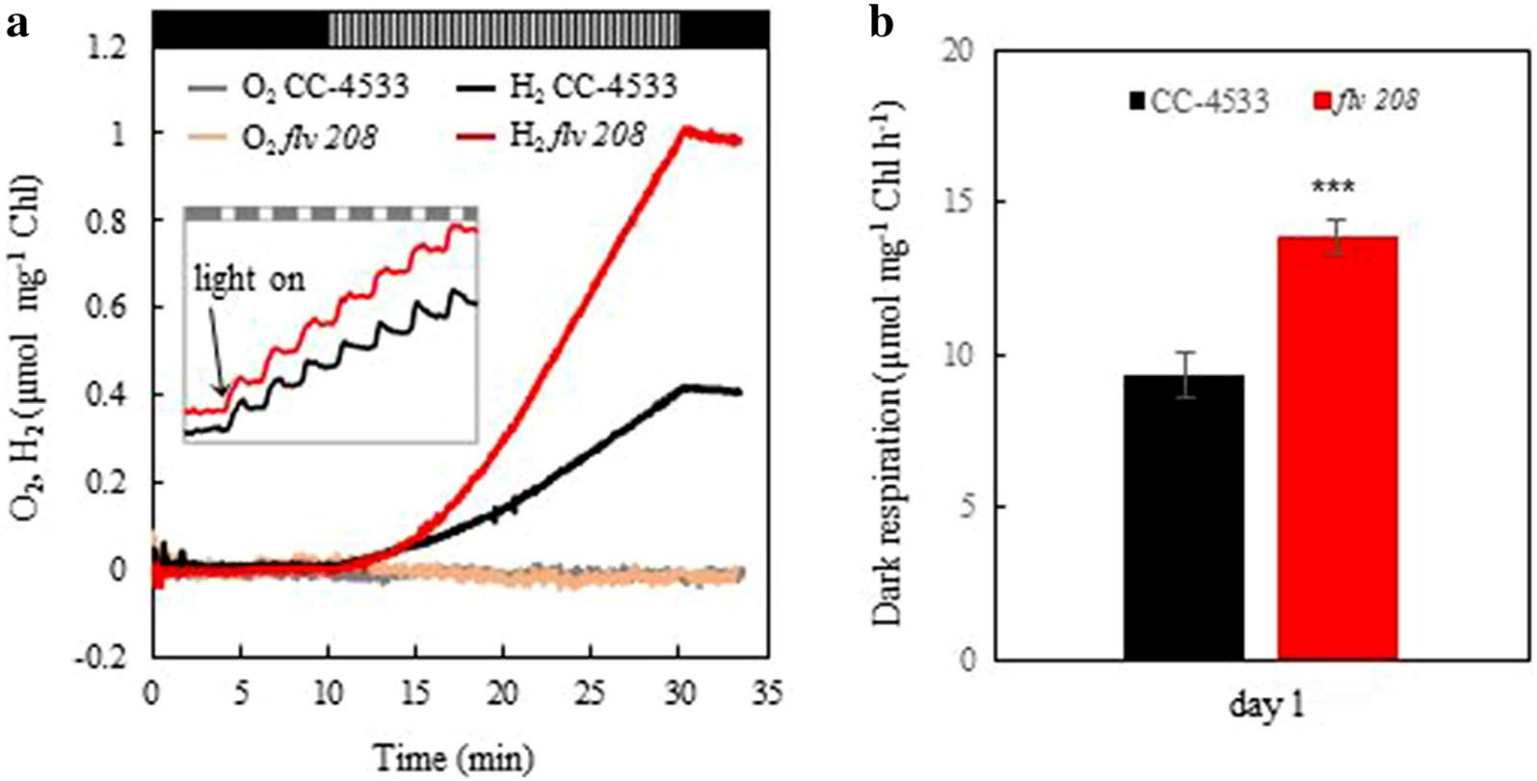
Short-term H_2_ photoproduction under 1-s light/9-s dark pulse illumination protocol in *C. reinhardtii*. The CC-4533 and the *flv* 208 mutant cells were grown for 2 days at 50 µmol photons m^−2^ s^−1^ in TP medium bubbling with 3% CO_2_, transferred to a vial equipped with H_2_ and O_2_ sensors, flushed with Ar. The intensity of light pulses was around 250 µmol photons m^−2^ s^−1^. **a** H_2_ and O_2_ yields during 10-min dark anaerobic adaptation phase, 20-min H_2_ photoproduction phase and 3-min dark H_2_ uptake phase. **b** Dark respiration rate monitored just before induction of the H_2_ photoproduction. Experiments have been performed in 4 independent replicates, and exemplary measurements are presented in **a**. The values in **b** are the mean of all replicates (± SD). Statistical significance level: ***p < 0.001

Short-term H_2_ photoproduction was induced in the wild-type *C. reinhardtii* CC-4533 and *flv* 208 knockout mutant, deficient in FLVB [26] by applying the pulse illumination protocol [8] under photoautotrophic conditions. Two-day-old cultures (Chl 3 µg ml^−1^) were transferred from continuous light to the pulse illumination without any further chlorophyll (Chl) adjustment or additional culture handling, like harvesting and resuspension. For anaerobiosis treatment, the cultures were flushed with argon (Ar) for 2 min in the dark followed by another 8 min of dark incubation. H_2_ photoproduction was initiated by a train of 1-s white light pulses (intensity of 250 µmol photons m^−2^ s^−1^) applied on dark background every 9 s (hereafter, 1/9 pulse illumination protocol) and monitored for 20 min. The experimental setup is depicted in Additional file 1: Figure S1. This protocol maintains anaerobiosis and keeps CO_2_ fixation inactive [8]. H_2_ photoproduction was detectable already upon the first light pulse both in the CC-4533 and in the *flv* 208 mutant cultures (Fig. 1a, inset). During 20 min of the 1/9 pulse illumination protocol, the *flv* 208 mutant produced more than double amount of H_2_ (1.00 µmol mg^−1^ Chl) than CC-4533 (0.35 µmol mg^−1^ Chl). No O_2_ accumulation was observed, implying maintenance of anaerobiosis in both cultures throughout the H_2_ production phase (Fig. 1a). However, the absence of O_2_ in the medium cannot exclude the presence of low levels of intracellular O_2_ during the pulse illumination due to the water-splitting activity of PSII, which could not be detected by the O_2_ electrode. Importantly, the second *flv* mutant line, *flv* 791, demonstrated a similar increased H_2_ photoproduction yield (Additional file 1: Figure S2), suggesting that the observed phenotype is truly due to the mutation in the *flvB* gene.

The Chl content and PSII efficiency (*F*_V_/*F*_M_) of the CC-4533 and *flv* 208 mutant did not differ significantly before applying the short-term 1/9 pulse illumination protocol (Additional file 1: Figure S3). However, the dark respiration measured in cultures before the induction of H_2_ photoproduction was higher in the *flv* 208 mutant as compared to CC-4533 (Fig. 1b), indicating that mitochondrial respiration compensates for the loss of FDPs in the mutant and contributes to the maintenance of anaerobiosis in the *flv* 208 culture during the H_2_ production phase. In line with a previous report, we observed strong H_2_ uptake in both cultures during the 9-s dark phases (Fig. 1a, inset) and during the 3-min dark phase upon termination of 1/9 pulse illumination protocol [8].

Next, we applied the 1/9 pulse illumination protocol to three (6 µg Chl ml^−1^)- and four (9 µg Chl ml^−1^)-day-old cultures. The CC-4533 cultures demonstrated a similar H_2_ photoproduction yield (0.35 µmol mg^−1^ Chl) during the third and fourth day as the 2-day-old culture (Fig. 1a and Additional file 1: Figure S4). The H_2_ photoproduction yield of 4-day-old *flv* 208 mutant cultures still was higher (0.45 µmol mg^−1^ Chl) compared to CC-4533; however, it was nearly twofold less than that at day 2 (Fig. 1a and Additional file 1: Figure S4). To clarify whether the high cell density at day 4 causes the decrease in H_2_ photoproduction in the *flv* 208 mutant, the Chl concentration of 2-day-old CC-4533 and *flv* 208 mutant cultures was set to the level of 4-day-old cultures. The concentrated 2-day-old cultures produced even more H_2_ than the original cultures with lower cell density (Additional file 1: Figure S5a). Moreover, a dilution of 4-day-old cultures did not affect the H_2_ photoproduction yield (Additional file 1: Figure S5b). These data indicate that the high cell density is not the reason for the decreased H_2_ photoproduction in the *flv* 208 mutant at day 4.

The PSII efficiency (*F*_V_/*F*_M_) and PSII effective yield, *Y*(II), of the CC-4533 and *flv* 208 cultures grown for two, three and  four days did not change over time and were the same in CC-4533 and *flv* 208 cultures (Additional file 1: Figure S3b, d). Thus, photoinhibition is not the reason for the decreased H_2_ production yield in older *flv* 208 cultures. It is important to note that dark respiration seems to decrease over time in the *flv* 208 mutant to the level of CC-4533 by day 4 (Additional file 1: Figure S3c). This suggests that in older cultures the loss of FDPs cannot be compensated by increased cooperation with mitochondrial respiration anymore (Additional file 1: Figure S3), and thus, H_2_ photoproduction in *flv* 208 decreases to wild-type levels. These data could indicate that in older cultures the O_2_ removal via FDPs is more important for the creation of microoxic niches than the cooperation with mitochondrial respiration. Our results suggest that setting a high cell density in young *flv* 208 mutant cultures could further improve the yield of H_2_ photoproduction by the 1/9 pulse illumination protocol.

**Fig. 2:**
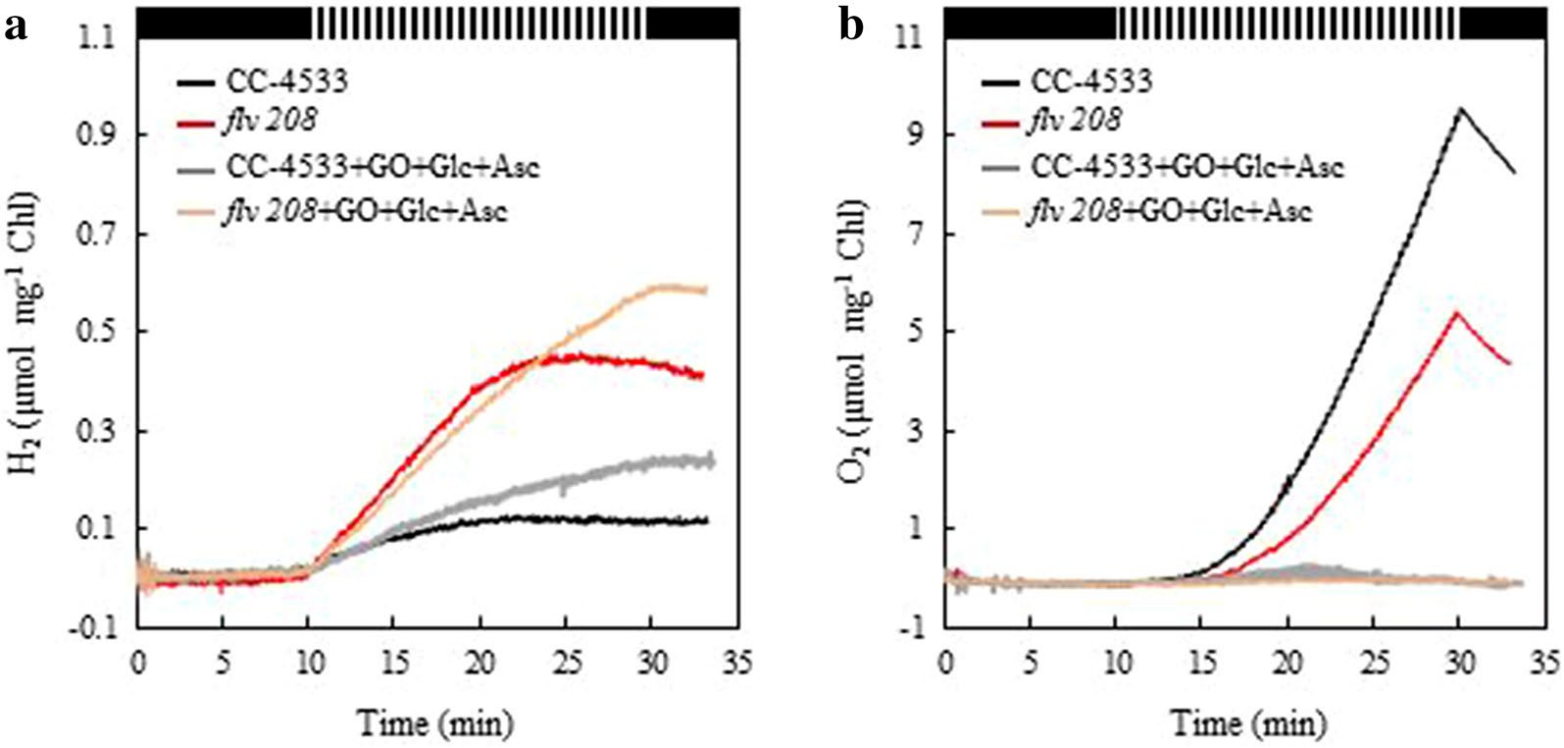
Short-term H_2_ photoproduction under 6-s light/9-s dark pulse illumination protocol in *C. reinhardtii* CC-4533 and in the *flv* 208 mutant. The other experimental conditions are the same as in Fig. 1. **a** H_2_ yield in the absence and presence of 10 U µl^−1^ glucose oxidase, 10 mM glucose and 1 mM ascorbate (GO + Glc + Asc). **b** Simultaneous monitoring of O_2_ yield in the absence and presence of GO + Glc + Asc. Experiments have been performed in at least 3 independent replicates, and exemplary measurements are presented

Next, we examined the effect of prolonged light pulses on H_2_ photoproduction in CC-4533 and the *flv* 208 mutant cultures (3 µg Chl ml^−1^). Under a 6-s light/9-s dark pulse illumination (hereafter, 6/9 pulse illumination) protocol the overall H_2_ production yield monitored in 2-day-old cultures was lower in both cultures as compared to the 1/9 pulse illumination protocol and completely stopped after 10 min of the pulse illumination (Fig. 2a). However, the decrease in H_2_ photoproduction under the 6/9 pulse illumination protocol was slightly more pronounced in CC-4533 (0.10 µmol mg^−1^ Chl compared to 0.35 µmol mg^−1^ Chl recorded under 1/9 pulse illumination) than in the *flv* 208 mutant (0.40 µmol mg^−1^ Chl compared to 1.00 µmol mg^−1^ Chl recorded under 1/9 pulse illumination). It is worth noting that the maximum specific H_2_ production rate of both cultures was more than twofold higher under the 6/9 than that under the 1/9 pulse illumination protocol (Additional file 1: Figure S6a). Furthermore, the *flv* 208 mutant exhibited a twice as high maximum H_2_ production rate compared to CC-4533 under both illumination protocols. Interestingly, the H_2_ production rates under the 1/9 pulse illumination protocol increased during the first 10 min of pulse illumination and remained stable at maximal rates during the following 10 min of pulse illumination (Additional file 1: Figure S6b). Importantly, during application of the 6/9 pulse illumination protocol strong net O_2_ evolution was observed in both cultures (Fig. 2b), possibly reaching levels that inhibit hydrogenase activity. Moreover, the O_2_ level was higher in CC-4533 compared to the *flv* 208 mutant probably due to the increased dark respiration in *flv* 208.

In order to eliminate the inhibitory effect of O_2_ during the 6/9 pulse illumination protocol, H_2_ photoproduction was monitored in cultures supplemented with 10 U µl^−1^ glucose oxidase (GO), 10 mM glucose (Glc) and 1 mM ascorbate (Asc). Indeed, both cultures remained anaerobic in the presence of the O_2_ scavenging system (Fig. 2b) and the yield of H_2_ photoproduction increased in CC-4533 (to 0.25 µmol mg^−1^ Chl) and even more strongly in the *flv* 208 mutant (to 0.60 µmol mg^−1^ Chl). It is important to note that in spite of the presence of GO the amount of H_2_ produced by the 6/9 pulse illumination protocol did not reach the level detected under 1/9 pulse illumination (Fig. 2a). This suggests that under the 6/9 pulse illumination an additional electron sink, besides the hydrogenases and FDPs, is activated and negatively affects H_2_ production. Activation of the CBB-cycle could be a likely candidate.

### Longer light pulses activate CO_2_ fixation

**Fig. 3:**
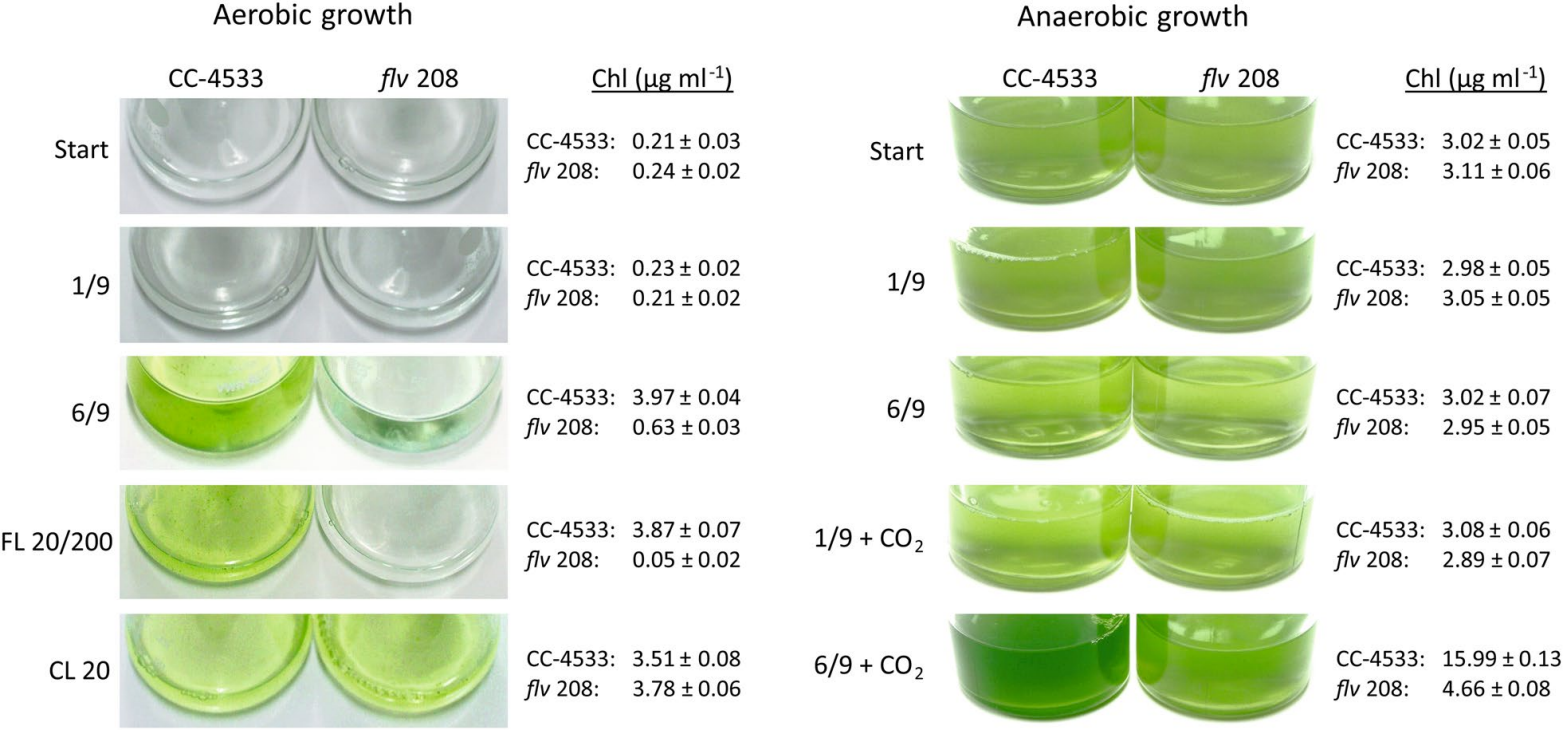
Growth phenotype of the *C. reinhardtii* wt CC-4533 and the *flv* 208 mutant cultures under different light illumination protocols. The aerobic (ambient air) and anaerobic (purged with Ar before the illumination) growth was monitored after 7 days of treatment under 1/9 and 6/9 pulse illumination protocols, fluctuating light protocol (20 µmol photons m^−2^ s^−1^ background light with 30 s 200 µmol photons m^−2^ s^−1^ illumination every 5 min, FL 20/200) and constant 20 µmol photons m^−2^ s^−1^ (CL) illumination with or without the addition of 3% CO_2_ to the headspace. Shown are examples for three independent replicates

Next, we studied whether activation of CBB-cycle and biomass accumulation occurs during the long-term light illumination protocols. The 1/9 pulse illumination consists of short light pulses and long enough dark intervals to avoid activation of the CBB-cycle, and therefore, cell growth is arrested [8]. Indeed, the CC-4533 strain and the *flv* 208 mutant did not show any change in Chl concentration during long-term (7 days) experiments under the 1/9 pulse illumination protocol (to settle anaerobic conditions, the headspace was purged with Ar for 10 min at the beginning of the protocol), implying the absence of biomass accumulation (Fig. 3). Importantly, under aerobic conditions (ambient air, 0.04% CO_2_) the same protocol did not stimulate growth in the CC-4533 and *flv* 208 mutant during 7 days. Lower starting Chl under aerobic conditions was used to be able to compare results to previous observed phenotypes [22]. At the same time, the prolongation of light pulses from 1 to 6 s without changing the length of the dark phase still restricted the growth under anaerobic starting conditions in both cultures, even though the cultures presumably did not remain anaerobic as O_2_ accumulation was detected in short-term experiments (Fig. 2b). The 6/9 pulse illumination protocol under aerobic conditions stimulated a substantial growth of the CC-4533. Importantly, only a slight increase in the Chl amount was observed in the *flv* 208 mutant under aerobic conditions (Fig. 3, 6/9 pulse illumination). These long-term growth experiments together with the increased level of O_2_ observed during the short-term 6/9 pulse illumination protocol (Fig. 2b) strongly suggest that CO_2_ fixation is activated under 6/9 pulse illumination.

In order to avoid carbon limitation in the anaerobic cultures, 3% CO_2_ was added to the vials after establishment of anaerobiosis (purged with Ar) and the Chl concentration was monitored in the cultures treated with 1/9 or 6/9 pulse illumination protocols over a period of 7 days (Fig. 3). Neither culture showed any increase in Chl content under the 1/9 pulse illumination, whereas under the 6/9 pulse illumination protocol CC-4533 demonstrated a strong growth. Notably, the growth of the *flv* 208 mutant was only slightly enhanced. This strongly suggests that indeed the 6/9 pulse illumination protocol activates CO_2_ fixation; however, this process is compromised in the *flv* 208 mutant to some extent.

In order to validate the activation of CBB-cycle under 6/9 pulse illumination, we supplemented the cells with the CBB-cycle inhibitor glycolaldehyde (GA) (Additional file 1: Figure S7). Both cultures, CC-4533 and *flv* 208, demonstrated increased H_2_ photoproduction in the presence of GA, thus verifying the activation of CBB-cycle under elongated light pulses. Importantly, *flv* 208 still demonstrated increased H_2_ photoproduction compared to CC-4533. Thus, differences in the activation kinetics of the CBB-cycle are not the reason for the increased H_2_ photoproduction in *flv* 208 under 6/9 pulse illumination. Furthermore, the higher H_2_ photoproduction in CC-4533 in the presence of GA occurred at almost unchanged O_2_ levels verifying that the CBB-cycle indeed outcompetes the [FeFe]-hydrogenase [38].

It was previously reported that the FDPs, FLVA and FLVB enable growth of *C. reinhardtii* under fluctuating light conditions [26]. Therefore, in the following experiment we tested the growth of *flv* 208 under similar conditions. Although the *flv* 208 mutant exhibited a slight increase in the Chl amount under aerobic and anaerobic (supplemented with 3% CO_2_) 6/9 pulse illumination, the application of fluctuating light of 5 min 20 µmol photons m^−2^ s^−1^ and 30 s 200 µmol photons m^−2^ s^−1^ (FL 20/200, similar to [26]) did not result in any growth in the *flv* 208 mutant culture. The FL 20/200 light condition was shown to create a strong PSI acceptor-side limitation in the *flv* 208 mutant, causing cell death [26].

### Pulse-illuminated algae sustain long-term photoautotrophic H_2_ production

**Fig. 4:**
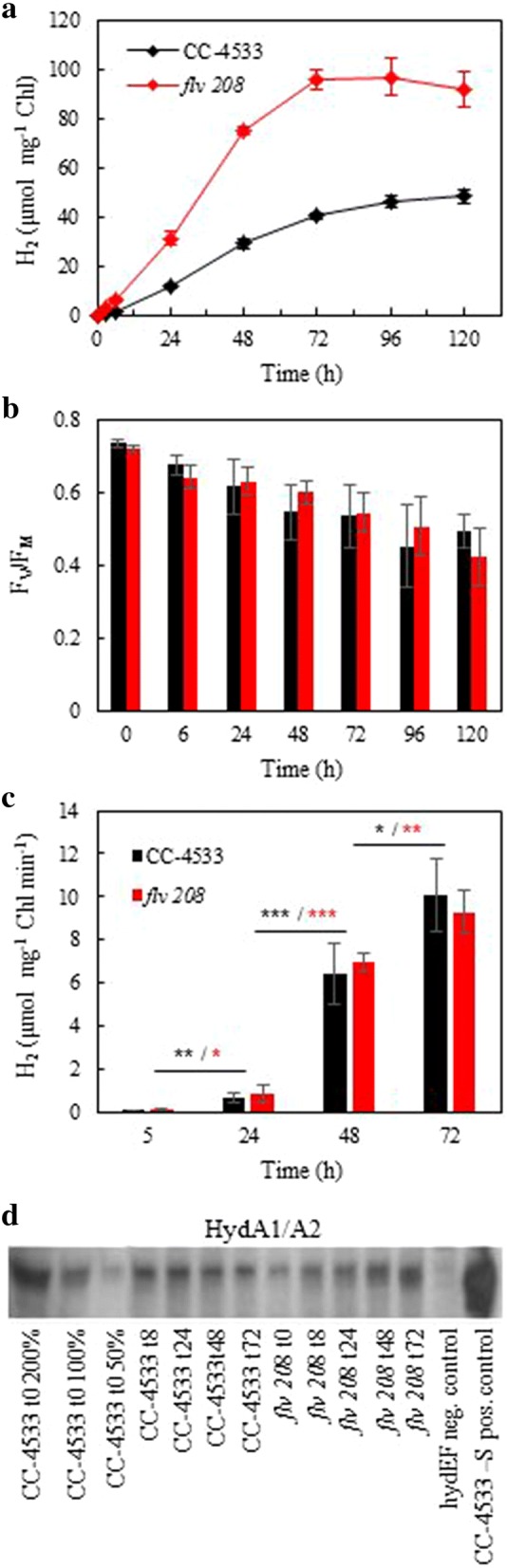
Long-term H_2_ photoproduction under the 1-s light/9-s dark pulse illumination protocol in *C. reinhardtii* CC-4533 and in the *flv* 208 mutant. The cells were grown in TP medium for 2 days in 50 µmol photons m^−2^ s^−1^ bubbling with 3% CO_2_ and were shifted to 1/9 pulse illumination without further adjustment (except 20-min purging with Ar). **a** Specific H_2_ production yield during 5 days of the experiment. **b** Photochemical efficiency of PSII (*F*_V_/*F*_M_) monitored during 5 days of H_2_ production under the 1/9 pulse illumination protocol. Values for **a** and **b** are mean of 6 independent replicates (± SD). **c** In vitro hydrogenase activity during 3 days of H_2_ production. Values are mean of 3 independent replicates (± SD). **b**, **c** No statistical significance between wt CC-4533 and *flv* 208. **c** Statistical significance levels between each time interval (5–24, 24–48 h and 48–72 h): *p < 0.05; **p < 0.01; ***p < 0.001. **d** Western blot analysis for the semiquantitative determination of HYDA1/A2 content, the first three lanes (200, 100, 50% of 0 h CC-4533) are for approximate quantitation of the proteins

For the long-term H_2_ photoproduction experiments, small gas-tight 75-ml vials were filled with 20 ml of 2-day-old photoautotrophic cultures grown in TP medium without any further adjustments [Chl content was usually around ~ 3 µg Chl  ml^−1^]. Since the 1/9 pulse illumination was more efficient for sustaining the process, we selected this protocol for long-term experiments. Both CC-4533 and *flv* 208 mutant cultures demonstrated H_2_ photoproduction during 3–4 days under the 1/9 pulse illumination protocol before H_2_ photoproduction ceased (Fig. 4a). In the long-term experiments, similarly to the short-term experiment (Fig. 1a), the *flv* 208 mutant produced almost double the amount of H_2_ (~ 90 µmol mg^−1^ Chl) than CC-4533 (~ 45 µmol mg^−1^ Chl) in 4 days.

In contrast to traditional nutrient deprivation approaches, the 1/9 pulse illumination protocol sustains H_2_ photoproduction in the presence of relatively active PSII. The *F*_V_/*F*_M_ decreased slowly from 0.75 to 0.45 within 5 days of H_2_ photoproduction (Fig. 4b), whereas the PSII activity decreases below 0.10 within 2 days of S-deprivation [35]. The protein levels of several key proteins involved in photosynthesis and respiratory processes were monitored over the course of the long-term H_2_ photoproduction (Additional file 1: Figure S8). This experiment showed that PSI core protein, PsaB, and RuBisCO, RbcL, levels were unchanged in both cultures by the end of the H_2_ production period. The level of the PSII core protein, PsbA, was reduced but not as drastically as shown for other H_2_ production protocols that are based on nutrient deprivations [36, 39–41]. This suggests that under the 1/9 pulse illumination protocol at least a portion of H_2_ may be produced via the PSII-dependent pathway. The NDA2 and PGRL1 proteins involved in cyclic electron transport [14, 42] showed an accumulation over time in both cultures. However, NDA2 accumulation was more pronounced in CC-4533 and PGRL1 accumulation was stronger in the *flv* 208 mutant (Additional file 1: Figure S8). The initial level of mitochondrial respiratory protein COXIIb was slightly higher in *flv* 208 than in CC-4533, which supports the high dark respiration rates in the mutant (Fig. 1b). The iron superoxide dismutase (FeSOD) protein level increased during the H_2_ production phase in both cultures. Notably, toward the end of the H_2_ production (*t*_72_ and *t*_96_) the accumulation level of FeSOD was higher in *flv* 208 than in CC-4533, indicating that FeSOD plays a role in compensating for the loss of FDPs in the mutant.

In order to clarify whether the high H_2_ photoproduction is due to an increased accumulation level or activity of hydrogenase, we performed immunoblotting using a specific antibody (Fig. 4d) and in vitro hydrogenase activity assay (Fig. 4c) in CC-4533 and the *flv* 208 mutant over 3 days of the 1/9 pulse illumination protocol. Both cultures, CC-4533 and *flv* 208, showed a gradual increase in the in vitro hydrogenase activity reaching about 10 µmol H_2_ mg Chl^−1^ min^−1^ at day 3 of the experiment. Importantly, CC-4533 and *flv* 208 demonstrated similar hydrogenase activities at each time point being consistent with [43]. The CC-4533 and *flv* 208 cells demonstrated an increase in HYDA1/A2 accumulation at 8 h under the 1/9 pulse illumination protocol (Fig. 4d).

After 8 h the HYDA1/A2 levels did not change much in CC-4533, while the levels further increased in the *flv* 208 mutant. This discrepancy between protein amount and enzyme activity levels in *flv* 208 could suggest partial inhibition of the [FeFe]-hydrogenases during the later H_2_ photoproduction phase. Importantly, HYDA1/A2 levels under the 1/9 illumination protocol were significantly lower compared to the S-deprivation control (Fig. 4d). However, as the in vitro hydrogenase activity does not differ between *flv* 208 and CC-4533, it cannot explain the increased H_2_ production in the *flv* 208 mutant.

### The effect of periodic recovery phases on long-term H_2_ photoproduction

**Fig. 5:**
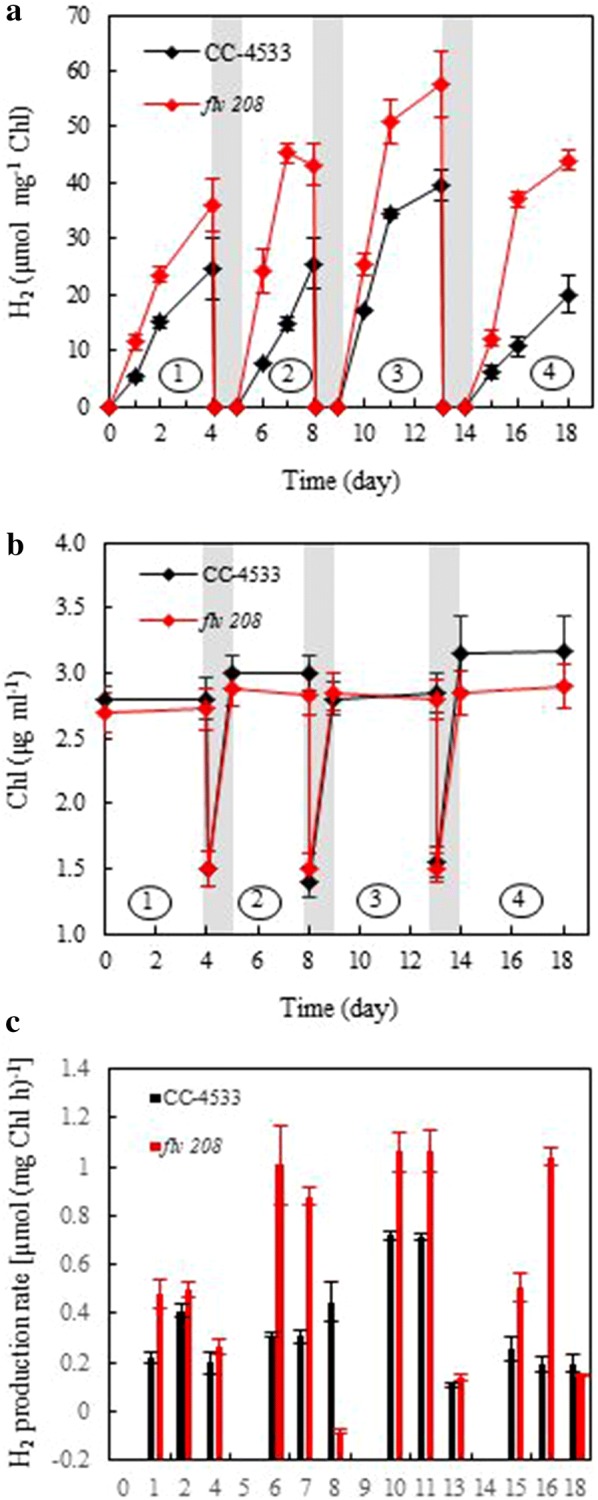
Effect of periodic 24-h growth recovery phases on long-term H_2_ photoproduction by the 1/9 pulse illumination protocol. *C. reinhardtii* CC-4533 and the *flv* 208 mutant were grown in TP medium for 2 days under 50 µmol photons m^−2^ s^−1^ bubbling with 3% CO_2_ before shifting to the H_2_ photoproduction conditions (Ar atmosphere and pulse illumination with 250 µmol photons m^−2^ s^−1^ pulses). The recovery phase includes 1:1 dilution with fresh TP medium and 24-h growth under 50 µmol photons m^−2^ s^−1^ bubbling with 3% CO_2_. **a** H_2_ photoproduction during 18 days. **b** Chl amount of the cultures during H_2_ photoproduction and recovery phases. **c** H_2_ production rates [µmol (mg Chl h)^−1^] during each cycle. These rates do not represent maximal H_2_ production rates as given in Additional file 1: Figure S6a. Values are mean of three independent replicates (± SD)

To study whether a prolonged H_2_ photoproduction process can be achieved by improving cell fitness through periodic and short-term exposure of the cells to air, the cells were shifted every 3–4 days from anaerobic 1/9 pulse illumination conditions to an aerobic recovery phase under continuous illumination (50 µmol photons m^−2^ s^−1^) and 3% CO_2_ bubbling for 24 h. During the recovery process, the cultures were diluted 1:1 with fresh TP medium and shifted to flasks. To restart H_2_ photoproduction small vials were refilled with the refreshed cultures and subjected to the 1/9 pulse illumination protocol. This process was successfully applied for 4 cycles equaling 18 days in total (Fig. 5a). Interestingly, in the later cycles H_2_ photoproduction started faster than in cycle 1, especially in the *flv* 208 mutant, indicating that the cultures remained primed for H_2_ photoproduction during the 24-h recovery phases. During the H_2_ photoproduction phases the CC-4533 and *flv* 208 cultures maintained a stable Chl amount (3 µg ml^−1^). After the 1:1 dilution with fresh TP medium (Chl 1.5 µg ml^−1^) both cultures were able to double their Chl amount (3 µg ml^−1^) within the 24-h recovery phases; thus, they did not require further Chl adjustment (Fig. 5b). The H_2_ production rates in between the recovery phases are presented in Fig. 5c.

### Photoautotrophic H_2_ production using a CBB-cycle substrate limitation protocol

**Fig. 6:**
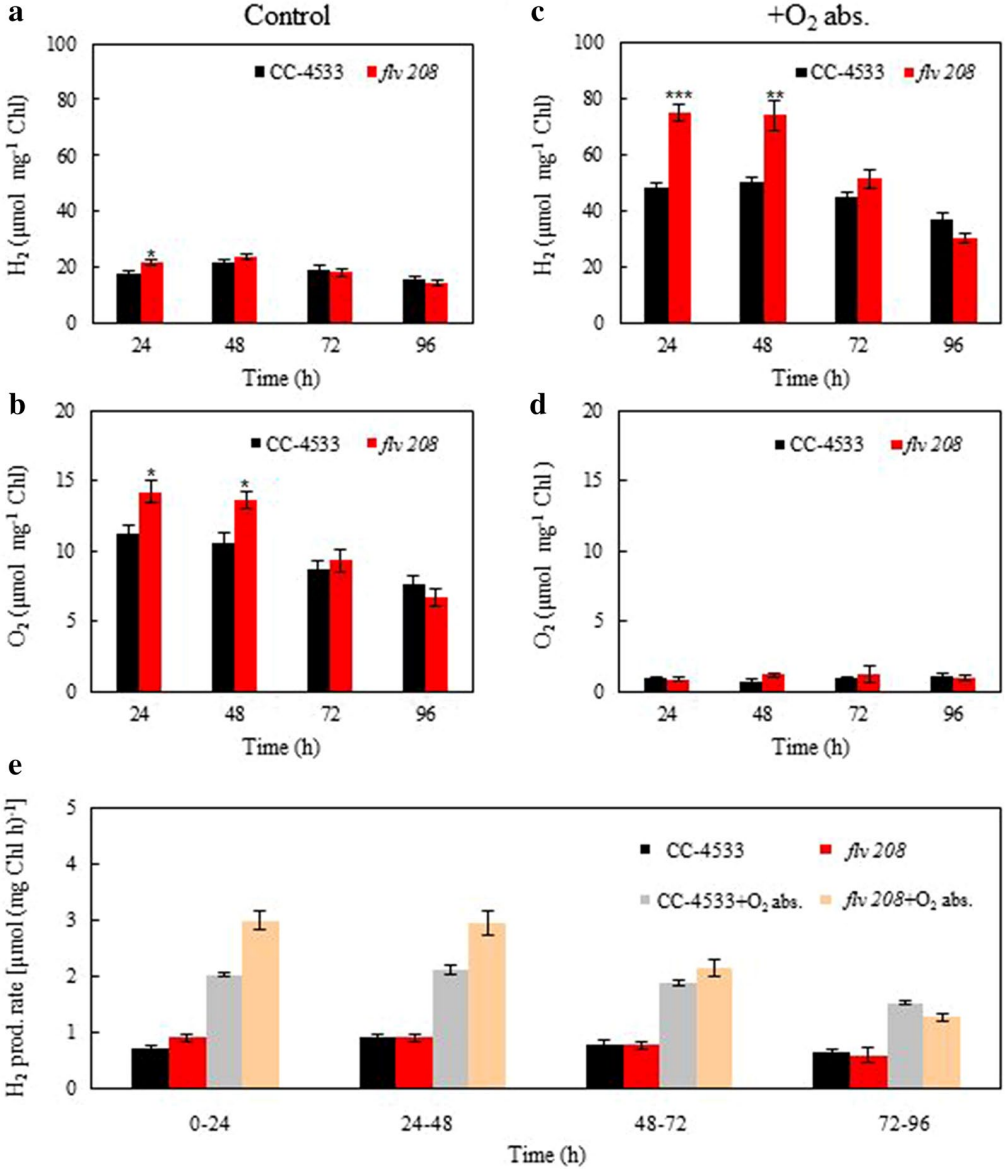
Long-term H_2_ photoproduction using a CBB-cycle substrate limitation protocol in *C. reinhardtii* CC-4533 and the *flv* 208 mutant. H_2_ photoproduction was induced by a 4-h dark anaerobic induction phase followed with constant 320 µmol photons m^−2^ s^−1^ illumination. Chlorophyll concentration was adjusted to ~ 50 µg Chl ml^−1^. **a**, **b** The daily H_2_ and O_2_ production (**a**, **b**; µmol mg Chl^−1^) in acetate-free HS medium during a 4-day experiment. **c**, **d** The effects of the iron-salt-based O_2_ absorbent on the H_2_ and O_2_ accumulation. **e** H_2_ production rates [µmol (mg Chl h)^−1^] during 96 h. Values are mean of 5 independent replicates (± SEM). Statistical significance levels: *p < 0.05; **p < 0.01; ***p < 0.001

The H_2_ production in CC-4533 and the *flv* 208 mutant was also analyzed when the cultures were subjected to another protocol that requires constant illumination, omits CO_2_ fixation and establishes anaerobiosis in the culture by limiting carbon availability [9]. The concentrated cultures (~ 50 µg Chl ml^−1^) in high salt medium (HSM) were incubated 4 h in dark anaerobic conditions and then constantly illuminated at 320 µmol photons m^−2^ s^−1^ for 4 days, and every day, the H_2_ level in the headspace was analyzed and was flushed with N_2_ gas. No significant difference (except on the first day, where *flv* 208 produced slightly more H_2_) between the *flv* 208 mutant and CC-4533 was observed during the 4 days of the H_2_ photoproduction experiment (Fig. 6a). Interestingly, O_2_ levels monitored in the headspace were higher during the first 2 days in the *flv* 208 mutant (Fig. 6b). The addition of an iron-salt based O_2_ absorbent to the headspace strongly reduced the O_2_ levels and elevated H_2_ photoproduction (Fig. 6d), which is in line with a previous study [9].

Importantly, the *flv* 208 mutant produced significantly more H_2_ during the first 2 days and then demonstrated a gradual decrease reaching the same level as CC-4533. This yielded in total ~ 230 µmol mg Chl^−1^ compared to ~ 170 µmol mg Chl^−1^ in CC-4533 within 4 days (Fig. 6c), which is about 3 times the H_2_ yield than in the 1/9 pulse illumination protocol during the same production period (Fig. 4a). It is important to note that the pulse illumination protocol was not carried out at optimal Chl concentration and light intensities and a direct comparison of the H_2_ production yields obtained by both protocols was not intended.

## Discussion

### FDPs are functioning simultaneously with hydrogenase at the onset of anaerobic induction

In nature, photosynthetic organisms are exposed to different stress conditions. To protect the photosynthetic apparatus from these environmental stress effects and to provide optimal energy and nutrient balance, phototrophs have developed a wide range of photoprotection mechanisms and alternative electron transport pathways. FDPs are a powerful electron sink, redirecting excess electrons downstream of PSI to O_2_ (called Mehler-like reaction). They were first described in cyanobacteria among photosynthetic organisms [21], and since then, intensive research revealed that FDP-mediated alternative electron transport is a universal pathway in photosynthetic organisms except for angiosperms [22, 24, 26, 44–47].

In our previous study, we suggested that under S-deprivation FDPs contribute to the rapid removal of O_2_ from the chloroplast, thus enabling fast establishment of anaerobic metabolism and H_2_ photoproduction [48]. Moreover, it was also shown that FDPs are down-regulated as soon as PSII activity becomes inhibited. Later it became clear that unlike in S-deprivation, under magnesium deprivation conditions both PSII and FDPs remain present for a longer period [41]. In line with this observation, it was proposed that FDPs are presumably involved in creating microoxic niches inside the chloroplast to enable [FeFe]-hydrogenase function during oxygenic photosynthesis [33]. These observations and assumptions suggest the simultaneous function of FDPs and the [FeFe]-hydrogenases and, thus, support the idea of eliminating the FDP-pathway as a new strategy to enhance H_2_ production in *C. reinhardtii* [43].

The 1/9 pulse illumination protocol [8] and the CBB-cycle substrate limitation protocol [9] both result in long-term photoautotrophic H_2_ production in CC-4533 and *flv* 208 cultures (Figs. 3 and 6). In contrast to S-deprivation, the PSII activity does not decrease dramatically under the 1/9 pulse illumination protocol (Fig. 4b) and the PsbA protein level only slowly declines during the H_2_ production period (Additional file 1: Figure S8), implying that at least during the first days, H_2_ is produced in a PSII-dependent manner. The electrons originating from water splitting at PSII during the 1-s light pulse are not used for CO_2_ fixation (the CBB-cycle remains inactive) as indicated by the growth limitation of the cultures (Fig. 3) and lack of CO_2_ uptake [8]. Instead, in CC-4533 the electrons originating from water splitting are used for H_2_ production, but many electrons are assumed to be “lost,” e.g., via the FDP-mediated pathway (Fig. 1a), which is a powerful electron sink at the onset of light under oxic conditions [24, 26]. Indeed, the mutant lacking FDPs, *flv* 208, produces substantially more H_2_ than CC-4533 under anaerobic (Fig. 1) or microoxic conditions (Fig. 2) by re-routing the electrons unused by FDPs toward H_2_ production. This is also corroborated by the higher initial maximal H_2_ production rates in the *flv* 208 mutant (Additional file 1: Figure S6a).

The nature of the electron acceptor for FDPs under anaerobic culture conditions (or microoxic conditions in the chloroplast) remains to be clarified. Can FDPs reduce O_2_ under very low O_2_ levels or is another electron acceptor involved? In *Synechocystis* sp. PCC6803, Flv3 and Flv1 homooligomers were shown to be involved in stress acclimation, however, without being involved in O_2_ photoreduction [49]. Since many bacterial FDPs are able to reduce NO [50, 51], a similar function is likely to occur in photosynthetic organisms as well. However, no K_M_ values of *C. reinhardtii* FDPs for O_2_ or NO are currently reported. It is not known whether intracellular NO concentrations reach levels that could sustain a significant electron transport. Nevertheless, the intracellular production of NO has been shown to modify the bioenergetics of chloroplasts in *C. reinhardtii* [52]. The possibility that in *C. reinhardtii* FDPs could be involved in the reduction of NO is especially interesting in the light of a recent study, showing the importance of NO signaling during H_2_ production conditions [53]. In addition to NO, the presence of another yet unidentified electron acceptor cannot be ruled out either.

A short-term increase in H_2_ photoproduction in the *flv* 208 mutant has been reported previously [43]. In the experimental setup used by [43] increased H_2_ photoproduction in the *flv* mutant compared to its parental strain occurs only after 40 s of illumination. Importantly, gross O_2_ evolution in this setup was determined indirectly from Δ*F*/*F*_M_ parameters, and the gross O_2_ uptake was calculated by subtracting the net O_2_ evolution from the calculated Δ*F*/*F*_M_-based gross O_2_ evolution. According to the authors, only when net O_2_ reaches a certain level (at about 50 s of illumination) the culture becomes aerobic and FDP-mediated O_2_ uptake occurs. Thus, the authors correlate the beginning of calculated O_2_ uptake with ceasing H_2_ photoproduction. In this scenario, [FeFe]-hydrogenases would be a first strong electron sink during photosynthetic induction after anaerobiosis and is later replaced by FDPs. In the current paper, we demonstrate that a train of as short as 1-s light pulses results in significantly higher H_2_ photoreduction in the *flv* 208 mutant. This implies an immediate competition between [FeFe]-hydrogenases and FDPs for photosynthetic electrons within 1 s of illumination (see increased H_2_ evolution in *flv* 208 upon the first 1-s pulse, Fig. 1a, inset). In contrast to [43] we demonstrate that FDPs function as an immediate fast and strong electron sink during the microoxic induction of photosynthesis as it is the case under aerobic culture conditions [25, 26].

Importantly, other photosynthetic alternative routes are also modified during the long-term pulse illumination protocol. In the later H_2_ production phase the NDA2 protein level increases (Additional file 1: Figure S8), which possibly correlates with increased electron transport from starch breakdown and fermentation toward H_2_ production [54, 55]. In addition, PGRL1-mediated cyclic electric transport becomes more important during the later H_2_ production phase [56] as indicated by the increased PGRL1 protein amount (Additional file 1: Figure S8).

One concern regarding the 1/9 pulse illumination protocol was whether the *flv* 208 mutant culture would remain anaerobic because of the missing FDP-mediated O_2_ photoreduction [26, 43, 48]. During the 1/9 pulse illumination protocol the O_2_ evolution at PSII and O_2_-consuming pathways, e.g., mitochondrial respiration, FDP- and PTOX-mediated O_2_ photoreduction presumably are in such a balance that the CC-4533 culture remains anaerobic (deduced from extracellular O_2_ levels, Fig. 1a), and consequently, the hydrogenase activity is not inhibited by photosynthetic O_2_ (Fig. 4c). Interestingly, the *flv* 208 culture also remains anaerobic (Fig. 1a) and the hydrogenase is as active as in CC-4533 (Fig. 4c). Our results suggest that the lack of FDPs was compensated by the increase in alternative O_2_-consuming pathways (Fig. 1b), likely mitochondrial respiration but possibly also other pathways like PTOX can contribute to the elimination of O_2_. This suggests that FDPs alone are dispensable for protecting HYDA1/A2 from inactivation by O_2_. This is in line with a previous report [43] that demonstrated that hydrogenase activity was not affected in a *flv* mutant during 2 min of illumination. Nevertheless, an involvement of FDPs in the protection of [FeFe]-hydrogenases in wild-type CC-4533 over a longer time period still cannot be completely excluded [33]. Accordingly, the slightly higher HYDA1/A2 protein amount in *flv* 208 but the same hydrogenase activity as in CC-4533 suggests possible hydrogenase inactivation in *flv* 208 after more than 3 days of 1/9 pulse illumination (Fig. 4c, d).

It appears that the substitution of FDPs by other respiratory pathways depends on the growth state of the culture because the dark respiration rate gradually declines reaching the level of CC-4533 in 4-day-old *flv* 208 cultures (Additional file 1: Figure S3c). This is observed together with the gradual decrease in H_2_ photoproduction to wild-type CC-4533 levels (Additional file 1: Figure S4). It is possible that in 4-day-old *flv* 208 cultures the intracellular O_2_ levels rise so high that an inhibition of hydrogenase activity occurs and the advantage in H_2_ photoproduction is eliminated.

The activities of the respiratory pathways seem to play a role in the CBB-cycle substrate limitation protocol as well. Growth in TAP for 3 days does not seem to activate the substituting respiratory pathways in the *flv* 208 culture, as indicated by the higher remaining O_2_ level in the headspace during the first 48 h (Fig. 6b). Again, in this case no advantage in H_2_ photoproduction as compared to CC-4533 is manifested (Fig. 6a). Incorporation of an O_2_ absorbent in the headspace of the culture largely enhanced hydrogenase activity under the CBB-cycle substrate limitation protocol [9]. Thus, when the O_2_ absorbent artificially removes excess O_2_, the H_2_ photoproduction in the *flv* 208 mutant increases as compared to CC-4533 (Fig. 6c, d).

Since the proposed function of FDPs is mainly relieving the excitation pressure upon a sudden increase in light intensity [24, 44, 45], from the first glance it is surprising to observe a strong increase in *flv* 208 H_2_ photoproduction using the CBB-cycle substrate limitation protocol (Fig. 6c). This protocol involves constant illumination [9], and thus, the effect of the *flv* knockout should not be as prevalent as under the pulse illumination protocol. However, due to the highly concentrated culture (~ 50 µg Chl ml^−1^) used in this protocol and the constant mixing, the individual cells likely face fast and drastic changes in light availability. This could explain the positive effect the absence of FDPs has on H_2_ production when applying the CBB-cycle substrate limitation protocol. On the other hand, a recent study suggests a prolonged function (at least 6 min) of FDPs in *C. reinhardtii* when carbon metabolism is impaired [25], or when *FLVA* and *FLVB* genes from the moss *Physcomitrella patens* were introduced into the *pgr5* mutant of *Arabidopsis* [57]. Additionally, in *Synechocystis* sp. PCC6803 the Flv1 and Flv3 proteins are able to drive a steady-state O_2_ uptake under high carbon conditions [32], thus suggesting that steady-state function of *C. reinhardtii* FDPs is possible.

### Elongation of the light pulses activates CO_2_ fixation and terminates H_2_ production under not carbon-limiting conditions

To possibly increase the H_2_ photoproduction yields, the light period was increased to 6 s, while the same 9-s dark period was applied during the pulse illumination protocol. During this elongated light period, more electrons originating from the water-splitting activity of PSII could be available for H_2_ photoproduction. Indeed, the initial maximal H_2_ production rates were higher under the 6/9 pulse illumination protocol than the 1/9 pulse illumination protocol (Additional file 1: Figure S6a). The increase in the light period induced a change in the PSII activity/respiration ratio in favor of PSII activity and leads to net O_2_ evolution. This, in turn, may have inhibited hydrogenase activity and the H_2_ production stopped after ~ 10 min of illumination (Fig. 2). In order to verify whether O_2_ removal would recover the long-term H_2_ production by the 6/9 pulse illumination protocol, O_2_ was enzymatically removed with the GO + Glc + Asc system (Fig. 2). Indeed, the cultures remained anaerobic and H_2_ photoproduction continued during the entire 20 min of the 6/9 pulse illumination protocol. Importantly, the monitored H_2_ photoproduction yield was substantially lower than that under the 1/9 pulse illumination protocol, suggesting the involvement of another active electron sink under the 6/9 pulse illumination. Since also the *flv* 208 mutant exhibited a lower H_2_ photoproduction yield under the 6/9 pulse illumination than the 1/9 pulse illumination, it is not possible that the FDP-mediated pathway accounts for this additional electron sink. More likely, the elongated light pulses activated CO_2_ fixation via the CBB-cycle. It is important to note that, even though short Ar purging is used to remove O_2_, presumably the cells have enough intracellular carbon storage to support CO_2_ fixation during the short-term experiments. This is supported by the fact that the CC-4533 cultures accumulate biomass under the long-term 6/9 pulse illumination protocol when cultures are supplemented with CO_2_ (Fig. 3). Under longer light pulses (6/9 pulse illumination) the CBB-cycle outcompetes H_2_ photoproduction before inhibition occurs [38], and the increased amount of O_2_ will later also inhibit the [FeFe]-hydrogenases. In this light, testing different light periods longer than 1 s but shorter than 6 s would be crucial to find the optimal period where the PSII activity/respiration ratio remains balanced, CO_2_ fixation stays inactive, but the number of electrons available for H_2_ photoproduction would be the maximum possible.

CO_2_ fixation is also activated and competes with the [FeFe]-hydrogenases for electrons in the *flv* 208 mutant as indicated by the lower H_2_ photoproduction yield under the 6/9 pulse illumination protocol with the addition of GO (compare H_2_ yield in Fig. 2a with Fig. 1a), the increased H_2_ photoproduction with the addition of GA (Additional file 1: Figure S7) and the (although small) growth (Fig. 3). The reduced growth of *flv* 208 is in line with the proposed function of FDPs activating CO_2_ fixation after the [FeFe]-hydrogenases stop functioning upon shifting from dark anaerobiosis to light oxic conditions [43]. However, also other effects are possibly involved in the reported impaired CO_2_ fixation in the *flv* 208 mutant, like the over-reduction of the PET chain and partial PSI inactivation as shown by the lack of fast P700 reoxidation upon changing light intensities [26]. These negative effects in the *flv* 208 mutant limit activation of CO_2_ fixation over longer time even under anaerobic conditions. Our results show that the [FeFe]-hydrogenases alone cannot efficiently activate CO_2_ fixation as previously proposed [58, 59]. Moreover, it is also conceivable that activation of CO_2_ fixation under anaerobic conditions depends not on a sequential function of [FeFe]-hydrogenase and FDPs [43], but on a simultaneous function of both pathways together.

### The pulse illumination protocol enables the combination of H_2_ production phases with recovery biomass accumulation phases

During the 1/9 pulse illumination protocol, CO_2_ fixation is disabled and instead the electrons are used for H_2_ photoproduction. If this process would continue, the culture would soon exhaust and be terminated as is the case for nutrient deprivation protocols (reviewed in [60]). To test whether CO_2_ fixation is disabled permanently or whether oxic photosynthesis and CBB-cycle can be resumed, the cultures were subjected to a recycling protocol. After 3–4 days of H_2_ photoproduction, the cultures were returned to aerobic growth conditions for 24 h before another H_2_ photoproduction phase was started. The cultures were able to rapidly resume efficient CO_2_ fixation and cell growth upon the change to oxic conditions (Fig. 5b). The RbcL protein level remains almost unchanged during the H_2_ photoproduction period, which enables a fast start of CO_2_ fixation (Additional file 1: Figure S8). This is in strong contrast to, e.g., the sulfur deprivation protocol, during which RbcL protein levels decline to zero within the first 48 h of H_2_ production [36, 41]. Remarkably, the H_2_ photoproduction in the later cycles started faster with higher yields within 24 h than during the first cycle (Fig. 5a), perhaps indicating higher [FeFe]-hydrogenase protein levels at the beginning of the later cycles. Another possible explanation could be that the PSII protein levels remain slightly diminished after the 24-h recovery phase, because this would accelerate the establishment of anaerobiosis.

The recovery experiment showed how rapidly and conveniently the culture can be switched from H_2_ production to biomass accumulation. The pulse illumination protocol still needs to be tested in large-scale production systems. It is worth mentioning that the prevention of CO_2_ fixation by applying light pulses and efficient O_2_ removal during dark phases in larger volume cultures is technically possible. In a suitable photobioreactor no culture handling like centrifugation or transfer to another culturing vessel would be necessary. The switch could be done by only changing CO_2_ availability and illumination rhythm. This is a common property with the CBB-limitation protocol developed by [9], and future investigations and engineering are necessary to compare and possibly combine these two protocols for maximum overall H_2_ production efficacy.

## Conclusion

This study demonstrates the immediate simultaneous function of FDPs and the [FeFe]-hydrogenases. We have also shown that the removal of FDPs as a competing electron sink results in the substantial increase in long-term H_2_ photoproduction via two promising induction protocols. We could also demonstrate that the elongation of light pulses during the pulse illumination protocol activates CO_2_ fixation and cell growth, which is why photosynthetic electrons are not available for H_2_ photoproduction anymore. The activation of CO_2_ fixation is disturbed in the *flvB* deletion mutant. Furthermore, we could show how a *C. reinhardtii* culture can be rapidly switched between H_2_ production and biomass accumulation phases by simply changing the illumination rhythm.

## Methods

### Algae growth and H_2_ production conditions

*Chlamydomonas reinhardtii* wild-type CC-4533 and the *flv* 208 knockout mutant (characterized in [26]) were analyzed for their H_2_ photoproduction performance. From actively growing *C. reinhardtii* cultures in TAP medium (photomixotrophic growth), experimental cultures were inoculated in TP medium (modified TAP medium, where acetate was replaced with HCl) and grown photoautotrophically under a 14-h photoperiod at 75 µmol photons m^−2^ s^−1^, at 25 °C, ~ 120 rpm shaking, and air was supplemented with 3% CO_2_ for bubbling.

Within 6 h from the start of the photoperiod, when photosynthesis is the most active and at day 2, 3 and 4 of the growth, the H_2_ photoproduction was analyzed with H_2_ and O_2_ microsensors (H2-NP and OX-NP, Unisense A/S) connected to a multichannel amplifier. In total, 19 ml culture was transferred into a gas-tight 23-ml GC vial, the electrodes were pierced inside through a Teflon-coated rubber septum, and the cells were sparged with argon (Ar) for ~ 2 min in the dark, followed by incubation in darkness for another 10 min. A train of 1-s or 6-s white LED light pulses (250 µmol photons m^−2^ s^−1^) interrupted by 9-s darkness was applied to the culture. The H_2_ and O_2_ levels were monitored by microsensors (Unisense) and the light protocol was controlled by OxyHydrogen software [8]. To eliminate residual O_2_, 10 mM Glc, 10 U µl^−1^ GO and 1 mM Asc were added to the culture during dark anaerobic incubation.

The long-term H_2_ photoproduction experiments were performed with 20 ml cell suspensions in 75-ml gas-tight vials under Ar atmosphere. The same protocols of light pulses as in the short-term experiments were provided by a growth chamber equipped with white LED sources (AlgaeTron AG 130-ECO, PSI). The vials were continuously shaken (~ 120 rpm), and H_2_ production yields were measured using a gas chromatograph (Clarus 500, PerkinElmer) equipped with a thermal conductivity detector and a molecular sieve 5 Å column (60/80 mesh). The total Chl content and hydrogenase activity were measured as described in [61]. For recycling after the 3–4-day-long H_2_ photoproduction experiment, the cultures from the 75-ml gas-tight vials were diluted 1:1 in TP medium and subjected to a 24-h growth regime under the same conditions as described earlier. Subsequently, the re-grown cultures were pipetted again into 75-ml gas-tight vials and H_2_ production was re-induced. This recycling of the culture was continued for a total of 4 cycles.

Another long-term H_2_ photoproduction protocol was also tested based on avoiding the CBB-cycle by substrate limitation [9]. Pre-grown 3-day-old *C. reinhardtii* cultures grown in TAP medium were harvested and transferred to HSM, and the Chl content was set to 50 µg Chl ml^−1^. H_2_ production was initiated by placing 30 ml culture in a 120-ml gas-tight serum bottle, sealed off with rubber septa. The gas phase was flushed with N_2_ gas for 10 min, followed by 4-h dark anaerobic incubation. Then, the cultures were kept at 26 °C for 96 h under T8 cool white fluorescent light tubes (Sylvania Luxline Plus), providing ~ 320 µmol photons m^−2^ s^−1^ PAR. To eliminate O_2_ from the headspace an iron-salt-based, non-cytotoxic O_2_ absorbent (O20TM; http://www.o2zero.com, 20 cc) was used. The H_2_ and O_2_ levels in the headspace were analyzed by GC. The serum vial was flushed with N_2_ gas daily after the determination of the gas concentrations.

### Photosynthetic activity

The DUAL-PAM-100 fluorometer (Walz, Germany) and DUAL-K25 quartz cuvette were used for the assessment of PSII parameters, based on Chl *a* fluorescence [62]. Cultures were measured at the indicated time points.

White light-saturating pulses (4000 µmol photons m^−2^ s^−1^, 500 ms) were used to measure the *F*_M_ and *F*_M_′ values. The PSII efficiency was estimated based on *F*_V_/*F*_M_ = (*F*_M_ − *F*_0_)/*F*_M_, and the effective yield of PSII was determined as *Y*(II) = (*F*_M_′ − *F*)/*F*_M_′. The O_2_ consumption rates in darkness were measured with a Clark-type O_2_ electrode (Oxygraph plus system, Hansatech, GB) at 25 °C.

### Hydrogenase assay protocol

In vitro hydrogenase activity was determined during long-term H_2_ photoproduction. The assay was carried out in 10-ml serum vials at 37 **°**C, and the reaction mixture (900 µl) consisted of 0,2% w/v Triton X-100 and 10 mM methyl viologen in 50 mM potassium-phosphate buffer (pH 6.9) and closed with a rubber seal. In total, 100 µl of anaerobic Na-dithionite was added to a final concentration of 100 mM. In total, 1000 µl of cells is taken from the culture vessel with a syringe and injected into the reaction mixture [61]. The H_2_ concentration in the headspace was measured by GC every 20 min, and the in vitro hydrogenase activity (µmol H_2_ mg^−1^ Chl min^−1^) was calculated.

### Western blot analysis

Cells were harvested and rapidly frozen in lysis buffer (50 mM Tris pH 8, 2% SDS, 10 mM EDTA, protease inhibitors from Sigma). After thawing, the total protein fraction was isolated and separated in a 12% SDS-PAGE with or without 6 M urea, transferred to a polyvinylidene difluoride membrane (Millipore) and blocked with 5% blotting grade blocker (Bio-Rad). The samples were loaded on equal protein basis as determined with Direct Detect^®^ infrared spectrometer (Merck) and visualized as control with Coomassie Brilliant Blue (Bio-Rad). Accumulation of PsaA, PsbA, FeSOD, COXIIb, RbcL and HydA1/A2 was analyzed by using specific antibodies (Agrisera). The PGRL1 and NDA2 antibodies were provided by Gilles Peltier (CEA Cadarache, Saint-Paul-lez-Durance, F-13108 France), and the FLVB antibody was described in [48]. As a secondary antibody, anti-rabbit horseradish peroxidase was used in 1:10,000 dilution and visualized with an enhanced chemiluminescence (ECL) kit (Amersham).

### Statistics

The presented data are based on at least three independent biological replications. Further datasets of exemplary shown measurements are available in the figshare repository (10.6084/m9.figshare.9862334.v1). When applicable, averages, standard deviations (SD) and standard errors (SE) were calculated. Statistical significance was analyzed using Student’s *t* test, and the significance level is presented as: *p < 0.05; **p < 0.01; ***p < 0.001.

## Supplementary information

**Additional file 1: Figure S1.** Schematic experimental setup of short-term pulse illumination experiments. In total, 19 ml of *C. reinhardtii* wt and *flv* mutant cultures grown in TP medium for 2 days in 50 µmol photons m^−2^ s^−1^ bubbling with 3% CO_2_ was withdrawn and subjected to less than 10-min darkness together with Ar purging before the 20-min pulse illumination experiments. **Figure S2.** Short-term H_2_ photoproduction under 1-s light/9-s dark pulse illumination protocol in two different knockout *flv* mutant lines. Cells were grown for 2 days at 50 µmol photons m^−2^ s^−1^ in TP medium bubbling with 3% CO_2_, transferred to a vial equipped with H_2_ and O_2_ sensors, flushed with Ar. The intensity of light pulses was around 250 µmol photons m^−2^ s^−1^. H_2_ yields during 10-min dark anaerobic adaptation phase, 20-min H_2_ photoproduction phase and 3-min dark H_2_ uptake phase in CC-4533, *flv* 208 and *flv* 791. Experiments have been performed in 3 independent replicates and are presented exemplary. **Figure S3.** Photosynthetic characteristics of *C. reinhardtii* wt and *flv* 208 mutant cultures grown in TP medium for 4 days in 50 µmol photons m^−2^ s^−1^ bubbling with 3% CO_2_. (a) Chl concentration (mg L^−1^) over a growth period of 4 days. The arrows show when the cultures were withdrawn for further treatments in the long- or short-term experiments. (b) Maximum quantum efficiency of PSII (F_V_/F_M_), (c) dark respiration and (d) effective yield of PSII (Y(II)) during 3 days of growth. Experiments have been performed in 5 independent replicates (± SD). (b, d) No statistical significance between wt CC-4533 and *flv* 208. (c) Statistical significance levels: *p < 0.05; ***p < 0.001. **Figure S4.** Short-term hydrogen photoproduction yield over 4 days of cultivation. H_2_ photoproduction is induced by the 1/9 pulse protocol in *C. reinhardtii* wt CC-4533 and the *flv* 208 mutant grown in TP medium for (a) 2 days, (b) 3 days and (c) 4 days under 50 µmol photons m^−2^ s^−1^ bubbling with 3% CO_2_. The curves depict H_2_ level during 10-min dark anaerobic phase, 20-min H_2_ photoproduction phase and 3-min dark H_2_ uptake phase. Experiments have been performed in 5 independent replicates and are presented exemplary. **Figure S5.** Short-term hydrogen photoproduction yield at different Chl concentrations. H_2_ photoproduction is induced by the 1/9 pulse illumination protocol in *C. reinhardtii* CC-4533 and the *flv* 208 mutant. (a) Two-day-old cultures (3 µg Chl ml^−1^) grown in TP medium under 50 µmol photons m^−2^ s^−1^ bubbling with 3% CO_2_ were concentrated (10 µg Chl ml^−1^) by centrifugation. (b) Four-day-old cultures (10 µg Chl ml^−1^) grown in TP medium in 50 µmol photons m^−2^ s^−1^ bubbling with 3% CO_2_ were diluted (3 µg Chl ml^−1^). Experiments have been performed in 3 independent replicates and are presented exemplary. **Figure S6.** H_2_ production rates in *C. reinhardtii* CC-4533 and *flv* 208 mutant. (a) Maximal H_2_ production rates under the 1/9 pulse illumination and 6/9 pulse illumination protocol. (b) H_2_ production rates during the 1/9 pulse illumination protocol. Cultures were grown 2 days under 50 µmol photons m^−2^ s^−1^ bubbling with 3% CO_2_. The maximal H_2_ production rates have been obtained within the last 5 min of pulse illumination for 1/9 pulse illumination and within the first 5 min of pulse illumination for 6/9 pulse illumination. Experiments have been performed in 4 independent replicates, and rates were calculated as mean of all replicates (± SD). Statistical significance level: **p < 0.01. **Figure S7.** Short-term H_2_ photoproduction under 6-s light/9-s dark pulse illumination protocol in *C. reinhardtii* CC-4533 and in the *flv* 208 mutant. The other experimental conditions are the same as in Fig. 1. (a and c) H_2_ yield in the absence and presence of 10 mM glycolaldehyde (GA). (b and d) Simultaneous monitoring of O_2_ yield in the absence and presence of GA. Experiments have been performed in 3 independent replicates, and exemplary measurements are presented. **Figure S8.** Immunoblot analysis of selected proteins from *C. reinhardtii* CC-4533 and *flv* 208 mutant grown under long-term 1/9 pulse illumination H_2_ photoproduction. The western blots shown here are representative of 3 biological replicates.

## Data Availability

The datasets generated and/or analyzed during the current study are available in the figshare repository (10.6084/m9.figshare.9862334.v1)

## Abbreviations

Asc: ascorbate
CBB: Calvin–Benson–Bassham
Chl: chlorophyll
FDPs: flavodiiron proteins
GO: glucose oxidase
Glc: glucose
LHCE: light energy to H_2_ energy conversion efficiency
H_2_: molecular hydrogen
HSM: high salt medium
NDA2: NADPH-dehydrogenase
PET: photosynthetic electron transport
PS: photosystem
TP: TRIS-phosphate medium
