# Supplementary material for: Elimination of the flavodiiron electron sink facilitates long-term H2 photoproduction in green algae
Source: Biotechnol Biofuels. 2019 Dec 5;12:280. doi: 10.1186/s13068-019-1618-1 (PMC6894204; doi:10.1186/s13068-019-1618-1)
Supplement: Supplementary file 1 — Additional file 1: Figure S1. Schematic experimental setup of short-term pulse illumination experiments. In total, 19 ml of C. reinhardtii wt and flv mutant cultures grown in TP medium for 2 days in 50 µmol photons m−2 s−1 bubbling with 3% CO2 was withdrawn and subjected to less than 10-min darkness together with Ar purging before the 20-min pulse illumination experiments. Figure S2. Short-term H2 photoproduction under 1-s light/9-s dark pulse illumination protocol in two different knockout flv mutant lines. Cells were grown for 2 days at 50 µmol photons m−2 s−1 in TP medium bubbling with 3% CO2, transferred to a vial equipped with H2 and O2 sensors, flushed with Ar. The intensity of light pulses was around 250 µmol photons m−2 s−1. H2 yields during 10-min dark anaerobic adaptation phase, 20-min H2 photoproduction phase and 3-min dark H2 uptake phase in CC-4533, flv 208 and flv 791. Experiments have been performed in 3 independent replicates and are presented exemplary. Figure S3. Photosynthetic characteristics of C. reinhardtii wt and flv 208 mutant cultures grown in TP medium for 4 days in 50 µmol photons m−2 s−1 bubbling with 3% CO2. (a) Chl concentration (mg L−1) over a growth period of 4 days. The arrows show when the cultures were withdrawn for further treatments in the long- or short-term experiments. (b) Maximum quantum efficiency of PSII (FV/FM), (c) dark respiration and (d) effective yield of PSII (Y(II)) during 3 days of growth. Experiments have been performed in 5 independent replicates (± SD). (b, d) No statistical significance between wt CC-4533 and flv 208. (c) Statistical significance levels: *p < 0.05; ***p < 0.001. Figure S4. Short-term hydrogen photoproduction yield over 4 days of cultivation. H2 photoproduction is induced by the 1/9 pulse protocol in C. reinhardtii wt CC-4533 and the flv 208 mutant grown in TP medium for (a) 2 days, (b) 3 days and (c) 4 days under 50 µmol photons m−2 s−1 bubbling with 3% CO2. The curves depict H2 level during 10- [file 13068_2019_1618_MOESM1_ESM.docx]

**Additional file**

**
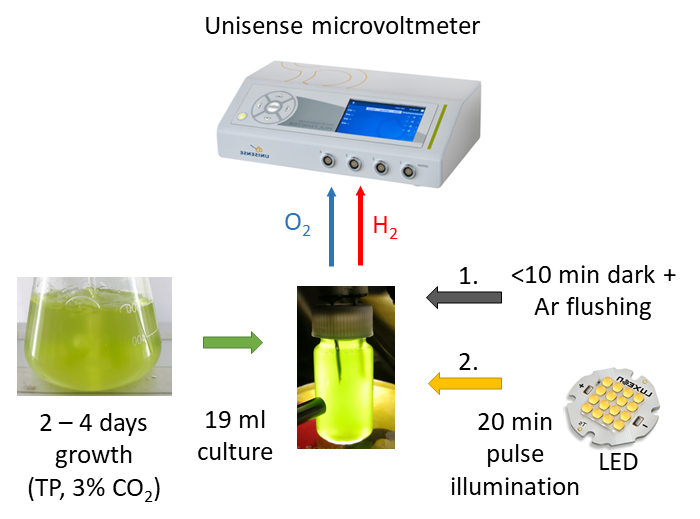
**

**S1**: Schematic experimental set-up of short-term pulse illumination experiments. 19 ml of *C. reinhardtii* wt and *flv* mutant cultures grown in TP medium for 2 days in 50 µmol photons m^-2^ s^-1^ bubbling with 3% CO_2_ were withdrawn and subjected to less than 10 min darkness together with Ar purging before the 20 min pulse illumination experiments.


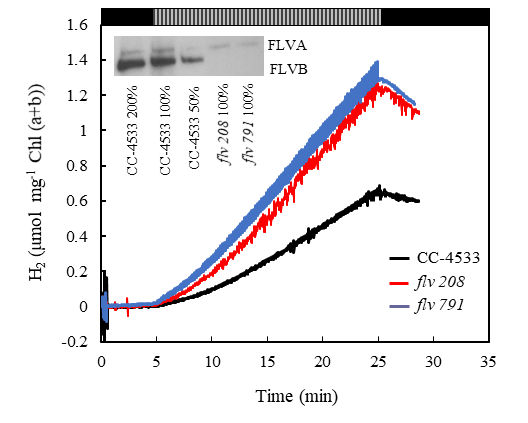


**S2**. Short-term H_2_ photoproduction under 1 s light / 9 s dark pulse illumination protocol in two different knock-out *flv* mutant lines. Cells were grown for 2 days at 50 µmol photons m^-2^ s^-1^ in TP medium bubbling with 3% CO_2_, transferred to a vial equipped with H_2_ and O_2_ sensors, flushed with Ar. The intensity of light pulses was around 250 µmol photons m^-2^ s^-1^. H_2_ yields during 10 min dark anaerobic adaptation phase, 20 min H_2_ photoproduction phase and 3 min dark H_2_ uptake phase in CC-4533, *flv* 208 and *flv* 791. Experiments have been performed in 3 independent replicates and are presented exemplary. The inset shows the verification of both *flv* mutant lines, *flv* 208 and *flv* 791, by immunoblot analysis.


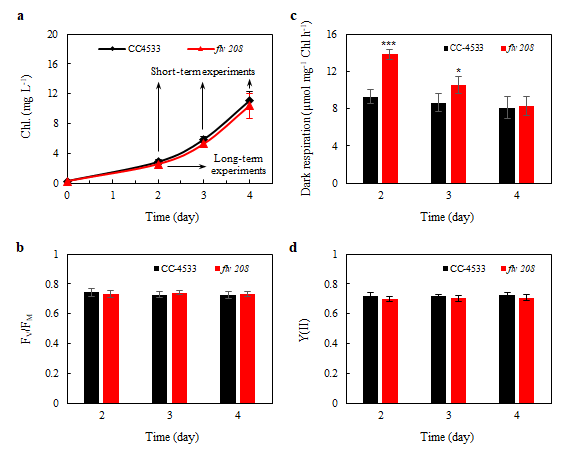


**S3**: Photosynthetic characteristics of *C. reinhardtii* wt and *flv* mutant cultures grown in TP medium for 4 days in 50 µmol photons m^-2^ s^-1^ bubbling with 3% CO_2_. (a) Chl concentration (mg L^-1^) over a growth period of 4 days. The arrows show when the cultures were withdrawn for further treatments in the long- or short-term experiments. (b) Maximum quantum efficiency of PSII (F_V_/F_M_), (c) dark respiration and (d) effective yield of PSII (Y(II)) during 3 days of growth. Experiments have been performed in 5 independent replicates (+/- SD). (b, d) No statistical significance between wt CC-4533 and *flv* 208. (c) Statistical significance levels: *p < 0.05; ***p < 0.001.


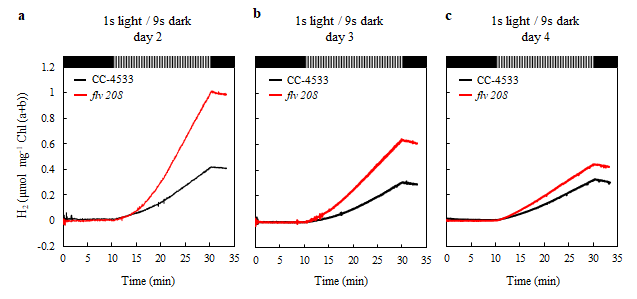


**S4**: Short-term hydrogen photoproduction yield over 4 days of cultivation. H_2_ photoproduction is induced by the 1/9 pulse protocol in *C. reinhardtii* wt CC-4533 and the *flv* 208 mutant grown in TP medium for (a) 2 days, (b) 3 days and (c) 4 days under 50 µmol photons m^-2^ s^-1^ bubbling with 3% CO_2_. The curves depicts H_2_ level during 10 min dark anaerobic phase, 20 min H_2_ photoproduction phase and 3 min dark H_2_ uptake phase. Experiments have been performed in 5 independent replicates and are presented exemplary.


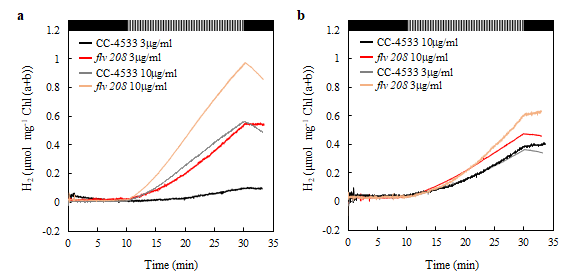


**S5**: Short-term hydrogen photoproduction yield at different Chl concentrations. H_2_ photoproduction is induced by the 1/9 pulse illumination protocol in *C. reinhardtii* CC-4533 and the *flv* 208 mutant. (a) 2 days old cultures (3 µg Chl ml^-1^) grown in TP medium under 50 µmol photons m^-2^ s^-1^ bubbling with 3% CO_2_ were concentrated (10 µg Chl ml^-1^) by centrifugation. (b) 4 days old cultures (10 µg Chl ml^-1^) grown in TP medium in 50 µmol photons m^-2^ s^-1^ bubbling with 3% CO_2_ were diluted (3 µg Chl ml^-1^). Experiments have been performed in 3 independent replicates and are presented exemplary.

**S6**: H_2_ production rates in *C. reinhardtii* CC-4533 and *flv* 208 mutant. (a) Maximal H_2_ production rates under the 1/9 pulse illumination and 6/9 pulse illumination protocol. (b) H_2_ production rates during the 1/9 pulse illumination protocol. Cultures were grown 2 days under 50 µmol photons m^-2^ s^-1^ bubbling with 3% CO_2_. The maximal H_2_ production rates have been obtained within the last 5 min of pulse illumination for 1/9 pulse illumination and within the first 5 min of pulse illumination for 6/9 pulse illumination. Experiments have been performed in 4 independent replicates and rates were calculated as mean of all replicates (+/- SD). Statistical significance level: **p < 0.01.


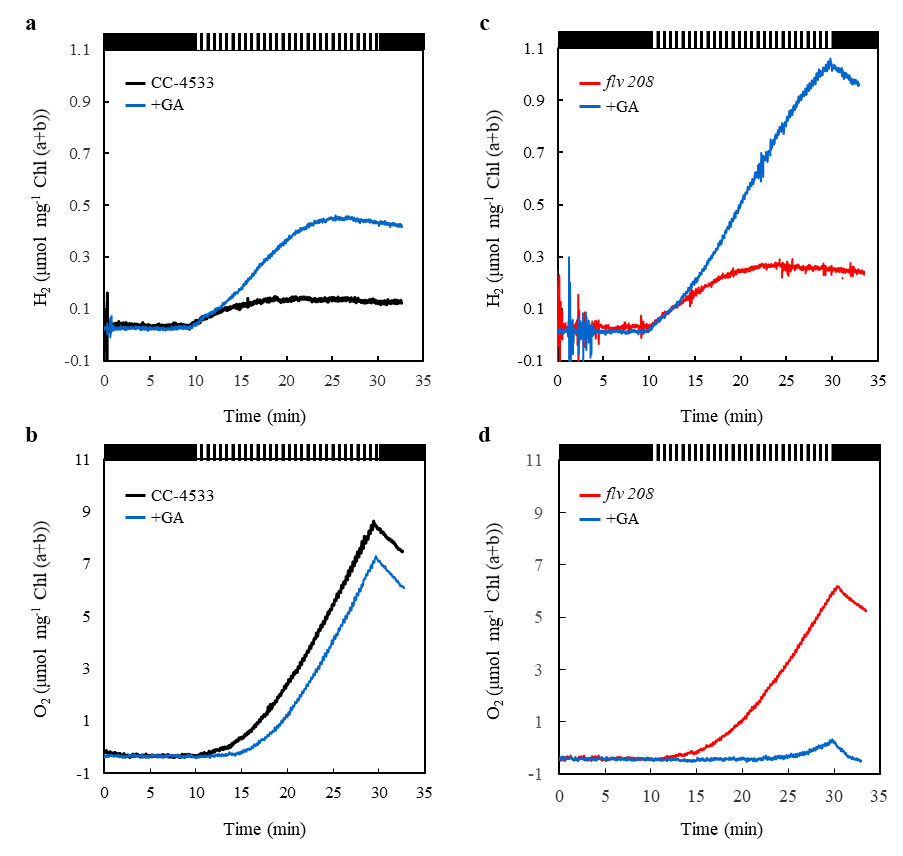


**S7**. Short-term H_2_ photoproduction under 6 s light / 9 s dark pulse illumination protocol in *C. reinhardtii* CC-4533 and in the *flv* 208 mutant. The other experimental conditions were the same as in Fig. 1. (a and c) H_2_ yield in the absence and presence of 10 mM glycolaldehyde (GA). (b and d) Simultaneous monitoring of O_2_ yield in the absence and presence of GA. Experiments have been performed in 3 independent replicates and exemplary measurements are presented.


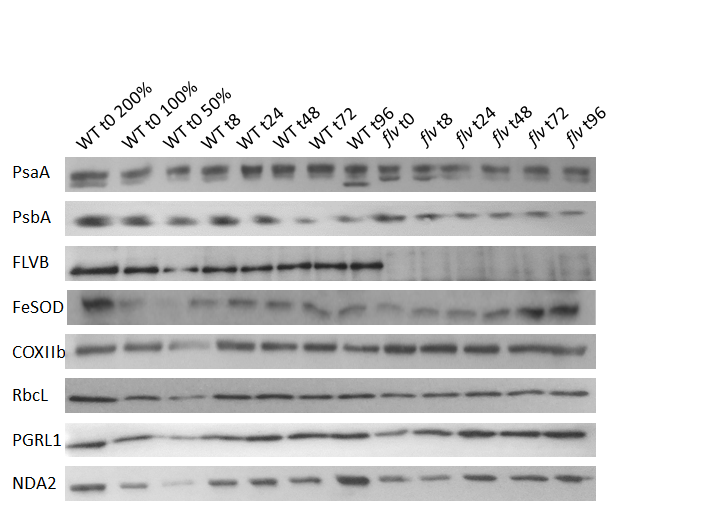


**S8**: Immunoblot analysis of selected proteins from *C. reinhardtii* CC-4533 and *flv* 208 mutant grown under long-term 1/9 pulse illumination H_2_ production. The western blots shown here are representative of 3 biological replicates.
